# The role of Mce proteins in *Mycobacterium avium paratuberculosis* infection

**DOI:** 10.1038/s41598-024-65592-2

**Published:** 2024-06-28

**Authors:** Rosemary Blake, Kirsty Jensen, Neil Mabbott, Jayne Hope, Joanne Stevens

**Affiliations:** The Roslin Institute and Royal (Dick) School of Veterinary Studies, University of Edinburgh, Edinburgh, UK

**Keywords:** Mycobacteria, *Mycobacterium avium* ssp *paratuberculosis*, MAP, Mammalian cell entry gene, Enteroids, Microbial-cell interaction, Microbiology, Molecular biology

## Abstract

*Mycobacterium avium* subspecies *paratuberculosis* (MAP) is the causative agent of Johne’s Disease, a chronic granulomatous enteritis of ruminants. MAP establishes an infection in the host via the small intestine. This requires the bacterium to adhere to, and be internalised by, cells of the intestinal tract. The effector molecules expressed by MAP for this purpose remain to be fully identified and understood. Mammalian cell entry (mce) proteins have been shown to enable other Mycobacterial species to attach to and invade host epithelial cells. Here, we have expressed Mce1A, Mce1D, Mce3C and Mce4A proteins derived from MAP on the surface of a non-invasive *Escherichia coli* to characterise their role in the initial interaction between MAP and the host. To this end, expression of *mce1A* was found to significantly increase the ability of the *E. coli* to attach and survive intracellularly in human monocyte-like THP-1 cells, whereas expression of *mce1D* was found to significantly increase attachment and invasion of *E. coli* to bovine epithelial cell-like MDBK cells, implying cell-type specificity. Furthermore, expression of Mce1A and Mce1D on the surface of a previously non-invasive *E. coli* enhanced the ability of the bacterium to infect 3D bovine basal-out enteroids. Together, our data contributes to our understanding of the effector molecules utilised by MAP in the initial interaction with the host, and may provide potential targets for therapeutic intervention.

## Introduction

*Mycobacterium avium* subspecies *paratuberculosis* (MAP) is a Gram positive, facultative intracellular, rod-shaped bacterium which is the causative agent of Johne’s Disease (JD). JD is a chronic gastric enteritis which affects ruminants across the world. Animals are typically infected with MAP during the first six months of life via the ingestion of contaminated milk and feed^1^. Other routes of infection include horizontal transfer from the environment and wildlife reservoirs such as rabbits, foxes and stoats^2,3^.

After ingestion, MAP bacteria travel to the small intestine of the animal and target specific cells in the intestinal wall to traverse and establish an infection. M cells are a specialised subset of epithelial cells that transcytose particulate antigens present in the intestinal lumen into the underlying gut-associated lymphoid tissues such as the Peyer’s patches^4^. These cells are also used by several enteric pathogens, such as *Salmonella*^5^ and prions^6^ to enter host tissues and establish host infection. Following their transcytosis by M cells, antigens or pathogens are subsequently acquired by mononuclear phagocytes to generate an appropriate immune response and/or eliminate the infection. A study conducted in mice suggested MAP, like other Mycobacteria, utilise M cells to transit across the intestinal epithelium^7^ which may then lead to infection of underlying immune cells such as macrophages. The infection of macrophages with MAP initiates a chronic inflammatory response which may lead to the formation of granulomas in the intestinal lining and clinical disease in 10% of infected animals^8,9^. However, currently there is very little known of the interaction between MAP and the bovine intestinal epithelium, including the role M cells may play in this.

Infections with MAP are chronic and may take between 2 and 5 years from the time of exposure until the animal shows clinical signs of disease, which typically presents as chronic diarrhoea and emaciation. Within the long subclinical period, MAP can be shed intermittently in the faeces, acting as a source of infection for other animals within the herd. Disease control can be difficult during this period since the burden on MAP in some infected animals may be below the level of detection for many diagnostic tests^10^. Due to the limited variety of diagnostic tests currently available for MAP, the identification and characterisation of MAP-specific proteins may aid the development of novel diagnostic and/or therapeutic targets.

Currently, the initial interaction between MAP and the host remains largely unknown. Studies in mice have suggested that MAP might use fibronectin attachment proteins (FAPs) expressed on its surface to target M cells^7^. Whether a similar mechanism is used to target these cells in ruminants is uncertain since FAP did not aid infection of bovine enterocytes. This raises speculation that MAP may express other adhesins on its surface which aid the infection of other host cell types of the intestine including enterocytes^11,12^ and goblet cells^13^.

Mammalian cell entry (*mce*) operons are highly conserved between Mycobacterial species. Latex beads or recombinant *E. coli* expressing Mce1A, Mce3C and Mce4A derived from *M. tuberculosis*,* M. bovis* or *M. leprae* have been observed to confer increased attachment and invasion of mammalian cells^14–17^. This suggests that these Mce proteins may play an important role in the initial attachment and invasion of host cells by several Mycobacterial species.

Data from a transposon mutagenesis study has shown that alterations to *mce1D* in MAP reduced the ability of the bacterium to infect bovine epithelial MDBK cells^18^. Due to the highly homologous nature of the *mce* operons between Mycobacterial species, we hypothesised that other *mce* genes would similarly increase the attachment and invasion of MAP to host cells. To test this hypothesis, the MAP-encoded *mce* genes *mce1A*,* mce1D*,* mce3C* and *mce4A* were recombinantly expressed in a non-invasive *E. coli* host and the effects of each gene on the ability of the bacteria to infect bovine epithelial MDBK cells, human monocyte-like THP-1 cells and bovine intestinal organoids (enteroids) were determined. Multiple cell lines were investigated to determine if these genes aid attachment, intracellular survival, or both, before being characterized in a physiologically representative model. The aim of this paper was therefore to identify if these selected *mce* genes were involved in the initial interaction between MAP and the host at the intestinal lining, with hopes of furthering our understanding of MAP pathogenesis and identifying potential therapeutic targets.

## Results

### Expression of *mce* genes in MAP upon exposure to acidic conditions

**Figure 1:**
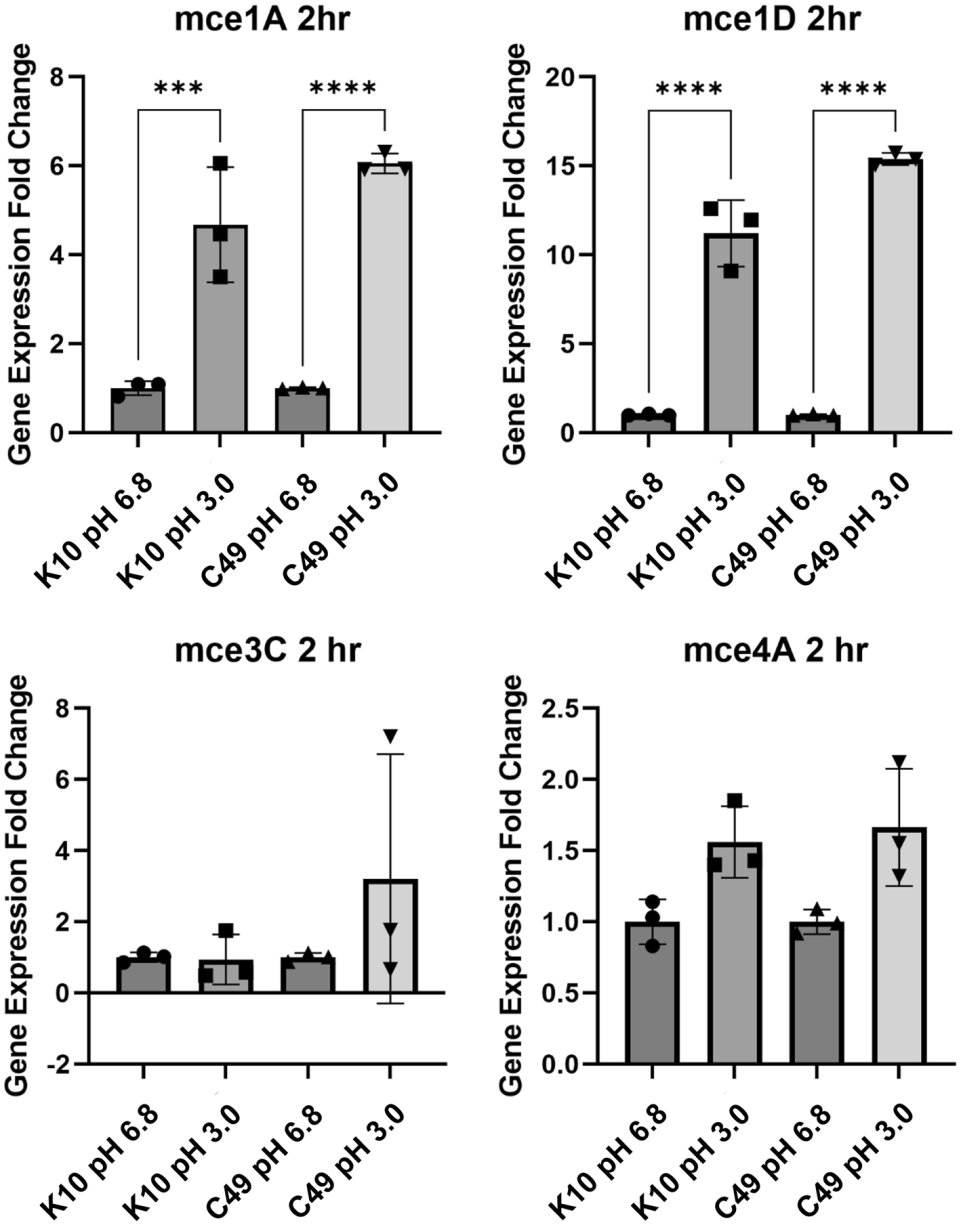
Regulation of MAP mce expression upon exposure to an acidic pH. The expression of mce genes was determined by RT-qPCR and calculated as fold change relative to the expression of gapdh and 1g2 as endogenous reference genes. Total RNA was isolated from 3 separate cultures of MAP K10 and C49 cultured to an OD_600_ 0.6 and pelleted to be re-suspended in standard 7H9 medium at pH 6.8 or pH3.0 7H9 medium. Data presented as the mean of the fold change in gene expression from 3 separate cultures ± SD. Statistatical analysis performed using a one-way ANOVA followed by a post hoc Dunnett’s test. *P* < 0.001 = ***; *P* < 0.0001 = ****.

Several adhesins expressed by MAP have been identified by quantifying gene upregulation in response to exposure to an acidic environment^19,20^. To this end, the expression levels of selected *mce* genes in MAP K10 and MAP C49 were determined following 2 h in vitro exposure in medium at pH 3.0. The fold-changes in *mce* gene expression in MAP exposed to pH 3.0 and control MAP cells exposed to medium at pH 6.8 were determined by RT-qPCR. Both strains of MAP significantly upregulated their expression of *mce1A* and *mce1D* after exposure to acidic pH, whereas the expression levels of *mce3C* and *mce4A* by either strain were unchanged compared to controls (Fig. 1). Thus, it is plausible that exposure to the decrease in pH in the gastro-intestinal tract following oral infection, upregulates the expression *map1A* and *map1D* in MAP to aid infection of host cells. Independent studies have reported the relative resistance of MAP to acidic pH as a high number of colony forming units (CFU) may still be recovered from treated cultures^21^. Here, although 10^8^ CFU/mL of MAP K10 were recovered 2 h post-exposure to pH 3.0, the number of viable bacteria was significantly reduced compared to control treated MAP (Supplementary Fig. 1). Furthermore, MAP C49 appeared to show a greater degree of sensitivity to acidity since a tenfold reduction in CFU/mL was observed post-treatment.

### Cloning, expression and purification of Mce proteins

**Figure 2:**
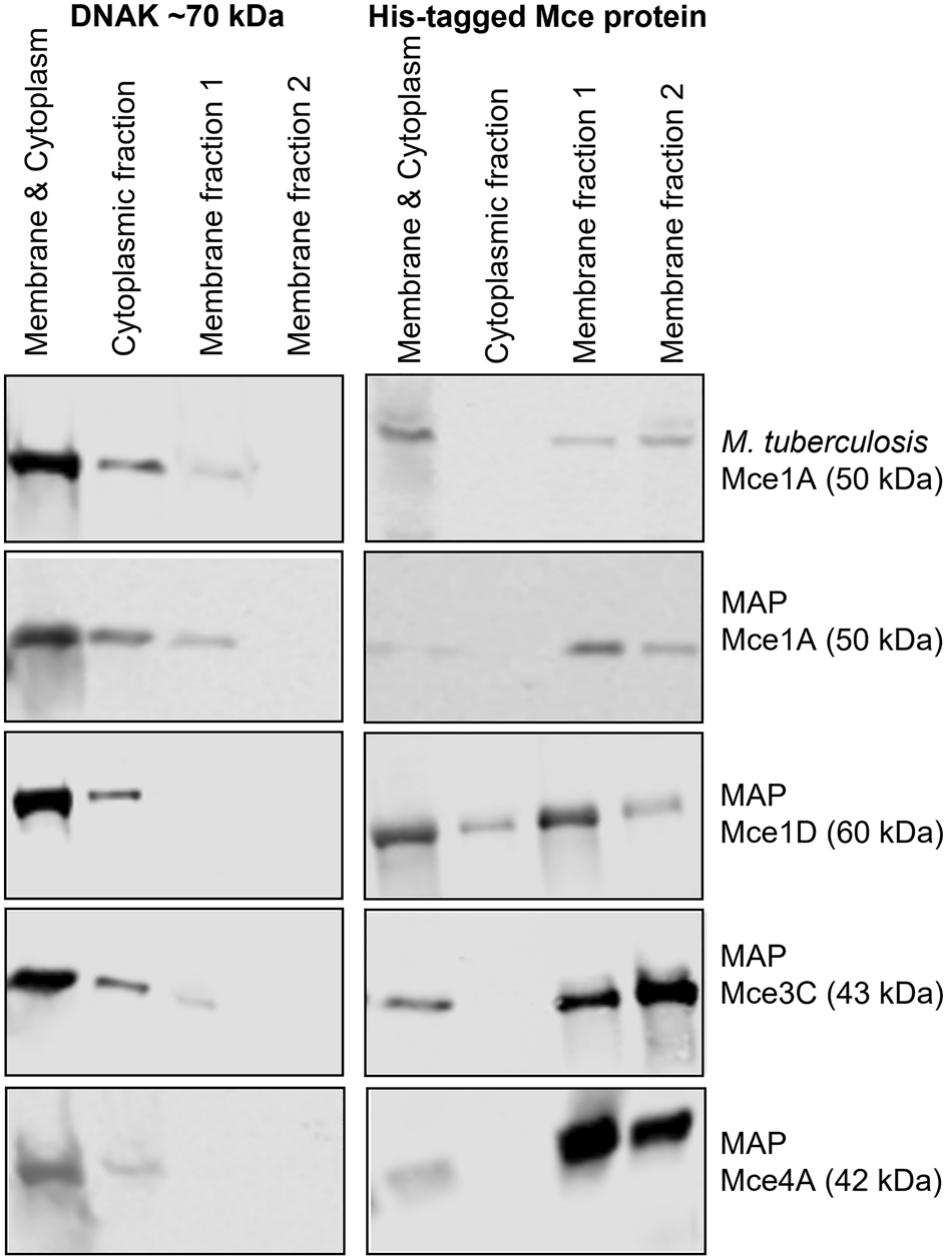
Western blot of *E. coli* strains expressing Mce protein after subcellular fractionation. *E. coli* clones were induced with 0.1 mM IPTG to produce their respective Mce protein for 2 h at 37 °C 180 rpm. The bacteria were then separated into fractions of the cell membrane and cytoplasm, the cytoplasm alone and two separate washes of the cell membrane. The fractions were separated by SDS-PAGE and electro-transferred to a nitrocellulose membrane. Rabbit monoclonal anti-His antibody was used to detect the His-tagged Mce protein to determine its location in the bacteria. Rabbit monoclonal anti-DNAK antibody was used as an *E. coli* cytoplasmic control. Images are cropped and each image represents an individual immunoblot. Whole gel images are found as supplementary Figures S3–S7.

The expression of the His tagged Mce proteins was studied in *E. coli* Rosetta 2 (BL-21). The proteins were detected by Western blotting using an anti-His-tag monoclonal antibody and were of the expected size, ranging from 43 to 60 kDa (Fig. 2). Bacterial suspensions were fractionated into cytoplasmic and cell membrane preparations to determine the expression site of each Mce protein. Immunostaining the blots with an antibody against DNAK, a known cytoplasmic protein in *E. coli*, was used to ensure separation of the bacterial cytoplasmic and cell membrane fractions (Fig. 2, left-hand panels). This analysis confirmed that there was variation in the level of Mce protein expression as normalised against the DNAK expression (Supplementary Figure S2). However, all Mce proteins were expressed in the membrane fractions of the *E. coli*, with only Mce1D detected in both the cytoplasmic and membrane fractions of the *E. coli* (Fig. 2, right-hand panels).

### Invasion of mammalian cells by recombinant *E. coli* expressing Mce proteins

**Figure 3:**
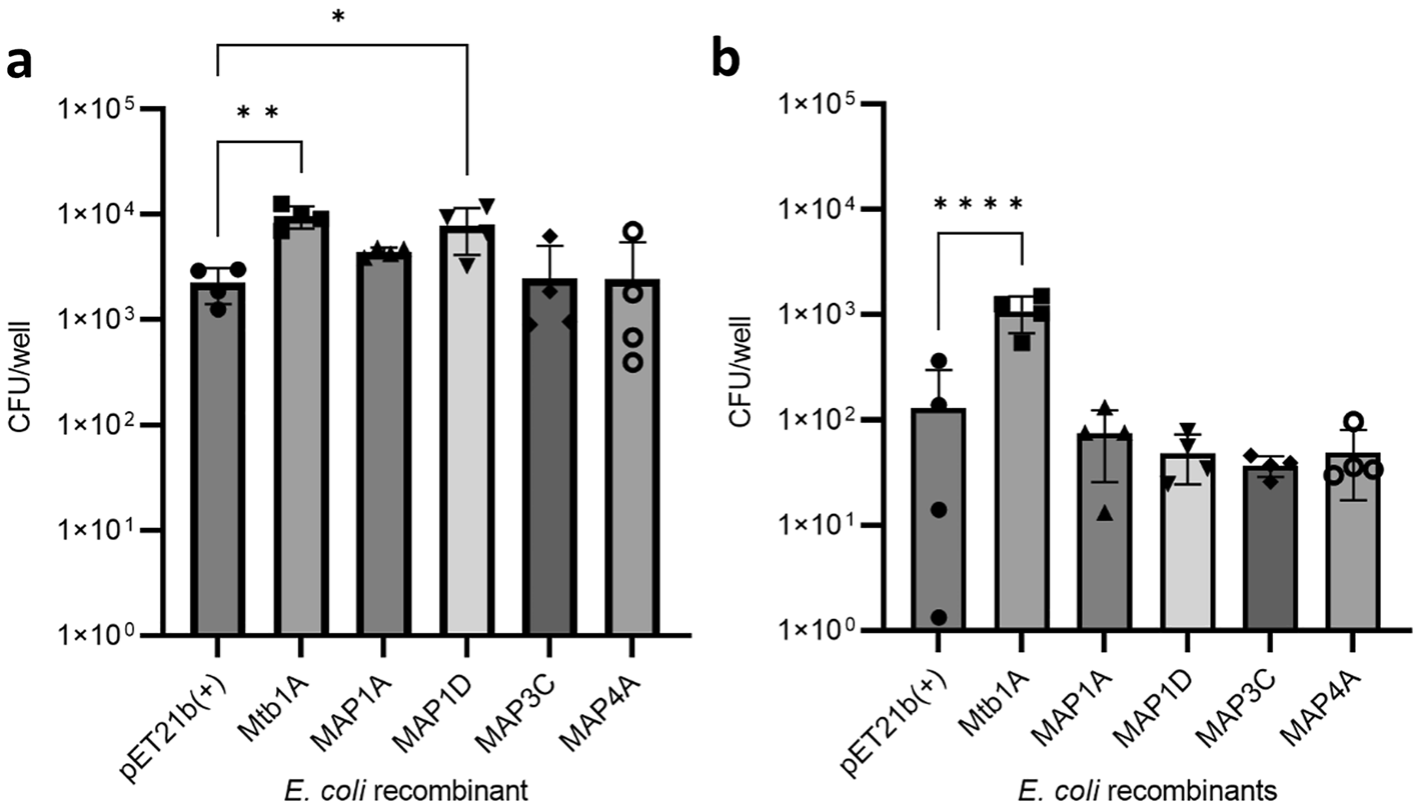
Attachment and survival of *E. coli* recombinants in MDBK cells. Mce protein expression was induced in *E. coli* recombinants with 0.1 mM IPTG for 2 h at 37 °C and used to infect MDBK cells at MOI 20. Cells were washed at 1 hpi and incubated for a further 1 or 5 h. Whole cell lysates were plated for CFU analysis which did not differentiate between attached or intracellular bacteria at 2 hpi (**a**); and at 6 hpi (**b**). Error bars presented as SEM of four biological replicates each performed with three technical repeats. Statistical analysis performed as a one-way ANOVA followed by a post hoc Dunnett’s test. *P* < 0.05 = *; *P* < 0.001 = **; *P* < 0.0001 = ***.

The expression of Mycobacterium-derived Mce proteins in *E. coli* did not significantly affect the viability of the bacteria compared to the pET21b(+) bacteria (Supplementary Figure S8). Next, the invasion of the MDBK bovine epithelial cell line by recombinant *E. coli* expressing Mce proteins was assessed by measuring bacterial burdens (CFU) in cell lysates at intervals after infection (Fig. 3). There was a modest but significantly greater abundance of *E. coli* expressing *M. tuberculosis* derived Mce1A (Mtb1A) or MAP-derived Mce1D (Map1D) in the cell lysates compared to the control pET21b(+) *E. coli* at 2 h post infection (hpi) (Fig. 3a). There was no increase in abundance for *E. coli* expressing MAP-derived Mce1A (Map1A), Mce3C (Map3C) or Mce4A (Map4A). In all proteins investigated, the number of *E. coli* had significantly diminished by 6 hpi, yet the abundance of recombinant bacteria expressing Mtb1A remained significantly elevated compared to cells exposed to pET21(+) control bacteria (Fig. 3b).

**Figure 4:**
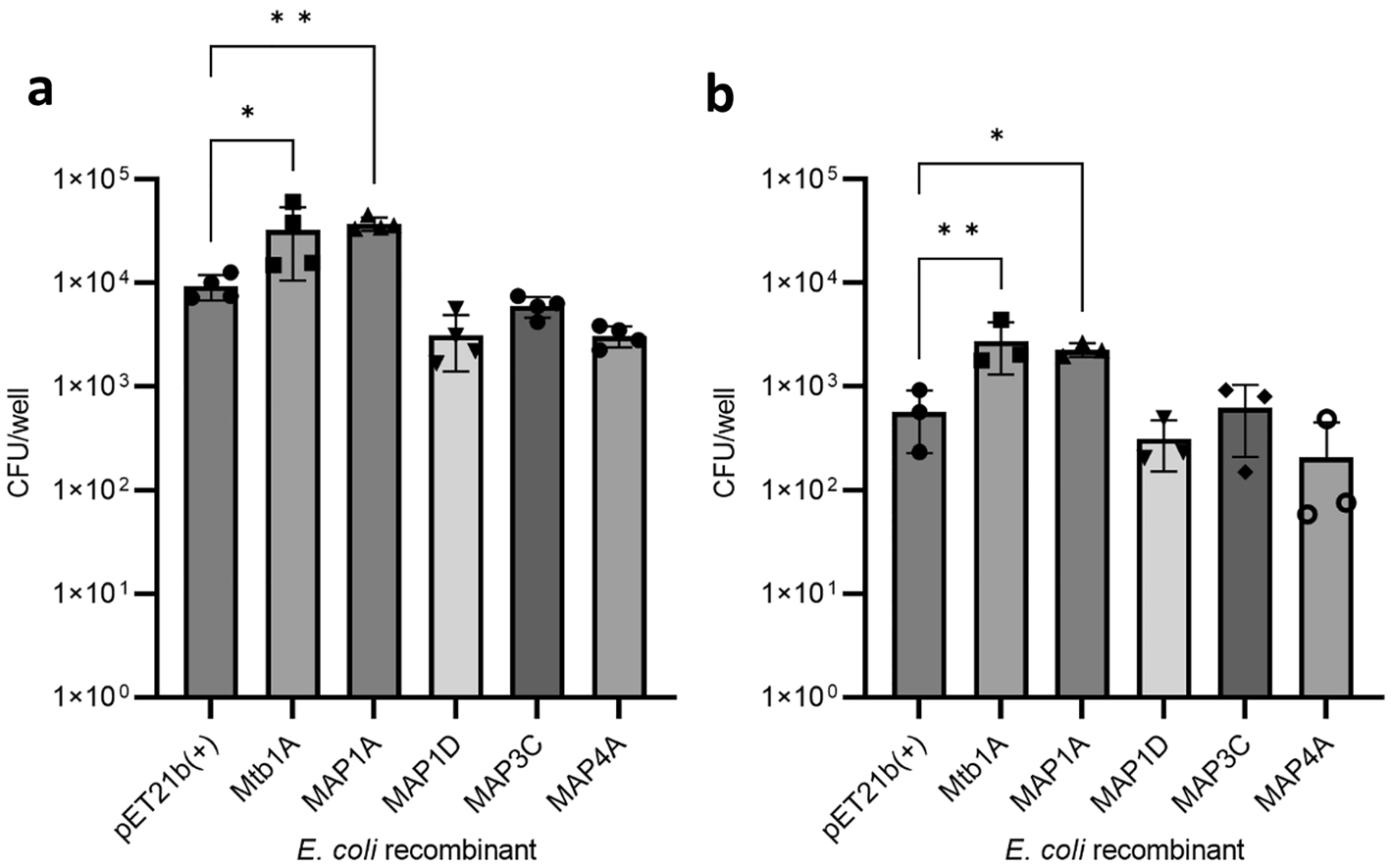
Uptake and survival of recombinant *E. coli* by THP-1 cells. Mce protein expression was induced in *E. coli* recombinants 0.1 mM IPTG for 2 h at 37 °C and used to infect THP-1 cells at MOI 10. Cells were incubated with medium containing 10 µg/mL gentamicin 1 hpi and incubated for a further 1 or 5 h. Cell lysates were plated for CFU analysis at 2 hpi (n = 4) (**a**); and at 6 hpi (n = 3) (**b**). Error bars presented as SEM of the specific number of biological replicates each performed with three technical repeats. Statistical analysis performed as a one-way ANOVA followed by a post hoc Dunnett’s test. *P* < 0.05 = *; *P* < 0.001 = **;

Next, human monocyte-like THP-1 cells were used to investigate the function of the MAP derived Mce proteins in the attachment and invasion of the bacteria into phagocytic cells. A modest yet significant increase was observed in the numbers of *E. coli* expressing Mtb1A and Map1A in the cell lysate compared to cells exposed to the pET21b(+) *E. coli* at 2 hpi (Fig. 4a). By 6 hpi the levels of *E. coli* expressing all proteins had reduced significantly, yet *E. coli* expressing Mtb1A and MAP1A remained significantly increased compared to the pET21(+) control (Fig. 4b).

**Figure 5:**
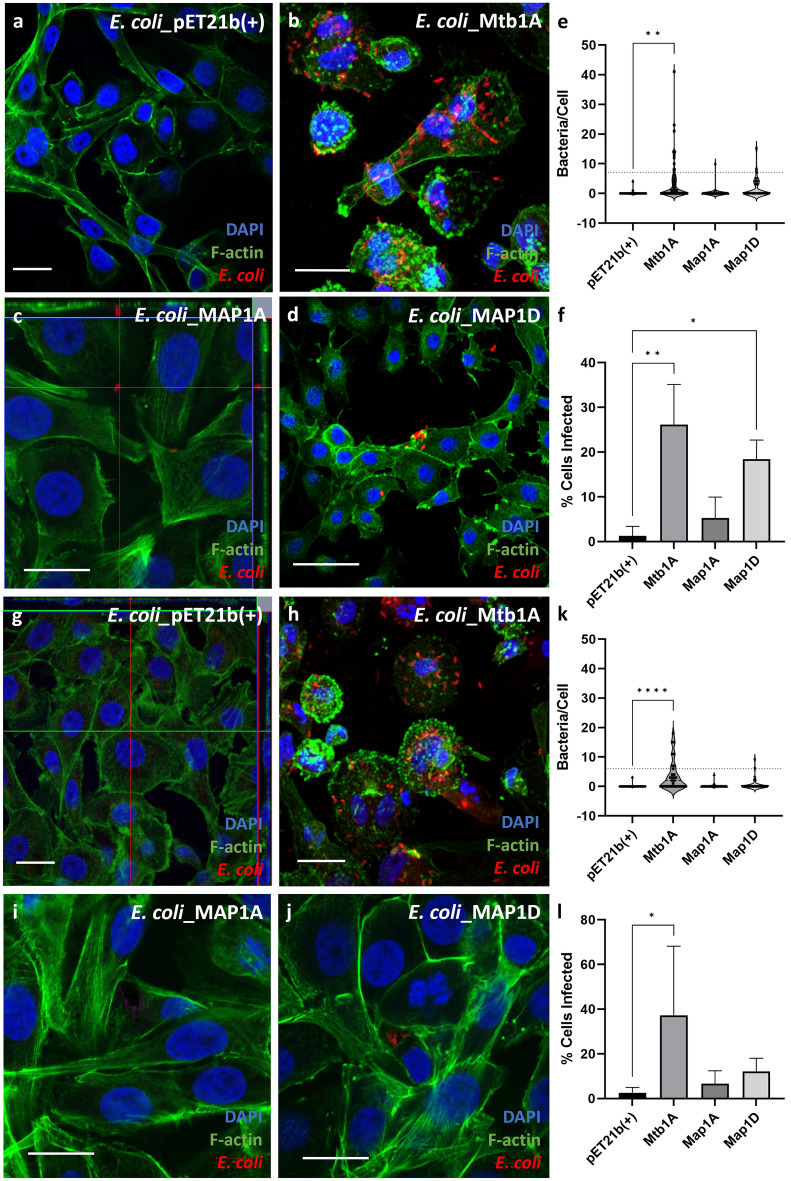
IF staining of infected MDBK cells with recombinant *E. coli*. The attachment and invasion of recombinant *E. coli* expressing Mce protein was visualised using IF staining and confocal microscopy at 2 (**a**–**d**) and 6 hpi (**g**–**j**). The cells were stained for nuclei (DAPI, blue), F-actin (Phalloidin, green) and anti-*E. coli* antibody (red). Scale bar = 10 µm. Using images collated from 2 ≤ biological replicates, the number of bacteria present in each cell were counted at 2 and 6 hpi (**e** and **k** respectively). Each data point on the graph represents an individual cell; the dotted line represents the average number of bacteria per infected cell at each time point, 7 (**e**) and 6 (**k**) bacterium for 2 hpi and 6 hpi respectively. The total proportion of infected cells were counted from images collated from 2 ≤ biological replicates at 2 and 6 hpi (f and l respectively). The data was analysed using a one-way ANOVA and post Hoc Dunnetts test. *P* < 0.05 = *; *P* < 0.001 = **.

The invasion of MDBK and THP-1 cells by recombinant *E. coli* expressing Mtb1A, Map1A and Map1D was monitored by confocal microscopy. In experiments using MDBK epithelial cells, there was no observed association of pET21b(+) *E. coli* with the cell (Fig. 5a), whereas *E. coli* expressing Mtb1A and Map1D were bound to the surface of the cells at 2 hpi (Fig. 5b and d). However, there was little observable association of Map1A *E. coli* to these cells (Fig. 5c). Mtb1A *E. coli* had a significantly greater number of bacteria per cell compared to pET21b(+) (Fig. 5e), and although a similar trend was shown for Map1D *E. coli*, this was not statistically significant. The proportion of cells associated with bacteria was significantly greater for both Mtb1A and Map1D *E. coli* compared to pET21b(+) at 2 hpi (Fig. 5f). At 6 hpi, more bacteria were observed attached to the cells only for Mtb1A *E. coli* compared to pET21b(+) *E. coli* (Fig. 5a–d), and this difference was statistically significant for both the number of bacteria per cell and the proportion of cells with cell surface-associated bacteria (Fig. 5k and l respectively).

Data shown in Fig. 5 are supportive of data reported in Fig. 3, and indicate that Mtb1A and Map1D aid both a greater number of bacteria to enter the cells and a greater number of cells to be infected at 2 hpi. Morphology changes in the MDBK cells were noted for Mtb1A *E. coli* infected cells at both 2 and 6 hpi, which is the only *E. coli* recombinant which had greater infection of MDBK cells at both time points (Figs. 3 and 5). Notably, the F-actin staining was altered for Mtb1A *E. coli* infected cells compared to the pET21b(+) cells.

**Figure 6:**
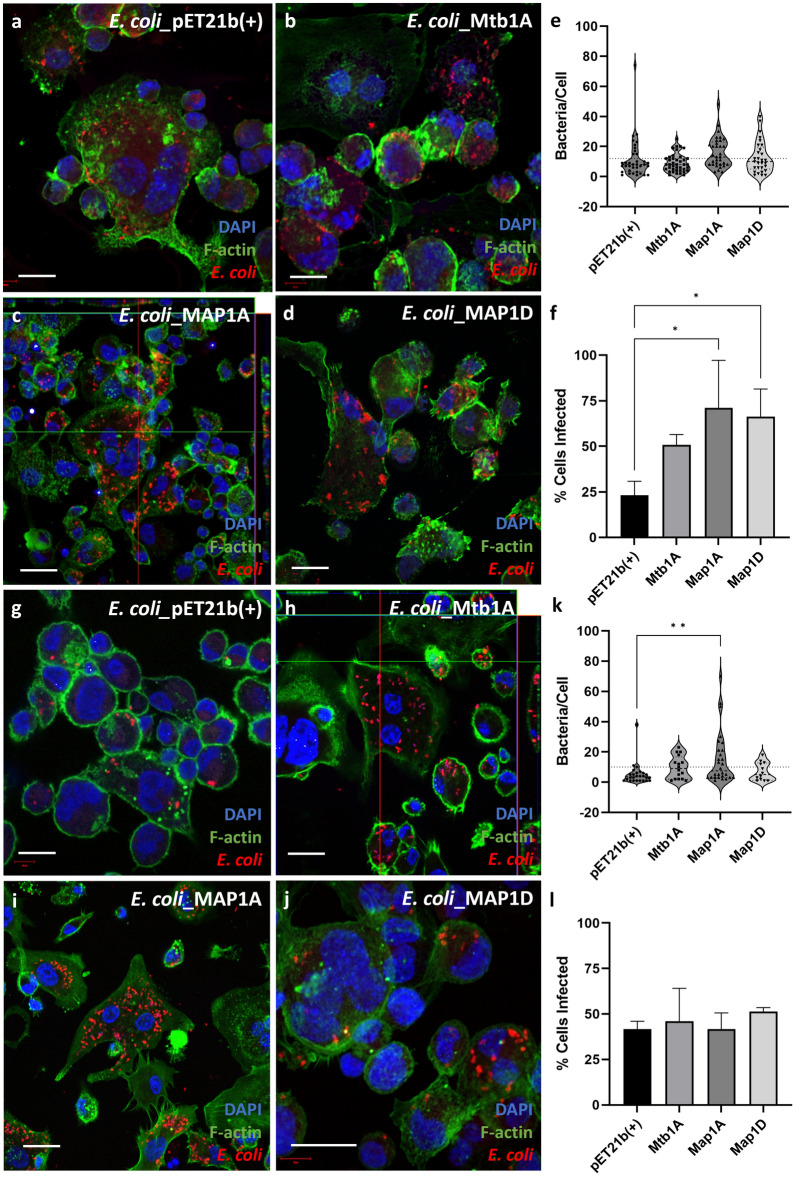
IF staining of infected THP-1 cells with recombinant *E. coli*. The attachment and invasion of recombinant *E. coli* expressing Mce protein was visualised using IF staining and confocal microscopy at 2 (**a**–**d**) and 6 hpi (**g**–**j**). The cells were stained for nuclei (DAPI, blue), F-actin (Phalloidin, green) and anti-*E. coli* antibody (red). Scale bar = 10 µm. Using images collated from 2 ≤ biological replicates, the number of bacteria present in each cell were counted at 2 and 6 hpi (**e** and **k** respectively). Each data point on the graph represents an individual cell; the dotted line represents the average number of bacteria per infected cell at each time point; The dotted line represents the average number of bacteria per infected cell at each time point, 12 (**e**) and 10 (**k**) bacteria for 2 hpi and 6 hpi respectively. The total proportion of infected cells were counted from images at 2 and 6 hpi (F and L respectively. The data is representative of the mean of 2 ≤ biological replicates ± SD and analysed using a one-way ANOVA and post Hoc Dunnetts test. *P* < 0.05 = *; *P* < 0.001 = **.

In experiments using phagocytic THP-1 cells, each of the *E. coli* recombinants was observed to be intracellular by 2 hpi. There were high numbers of bacteria per cell observed for all conditions (Fig. 6a–d), with no significant differences between the number of bacteria per cell (Fig. 6e). This is likely due to the fact THP-1 cells are phagocytic actively took up bacteria without aid of the Mce protein expressed on the surface of the *E. coli* (Fig. 6e). All *E. coli* expressing Mce protein showed a trend for a greater proportion of cells infected compared to the pET21b(+) control, but this was only significant for Map1A and Map1D (Fig. 6f). This differs from data shown in Fig. 4a as there was no increase in Map1D *E. coli* recovery from the THP-1 cell lysates from this infection. Therefore, it is likely that if Map1D does aid entry of THP-1 cells, it is unlikely to enhance the viability of these bacterium once they become intracellular.

By 6 hpi, only Mtb1A and Map1A *E. coli* had a high number of bacteria present in multiple cells (Fig. 6g–j) which was statistically significant for Map1A *E. coli* (Fig. 6i and k). It was only Mtb1A and Map1A *E. coli* conditions which had more than one infected cell with a bacterial load greater than 15 (Fig. 6k). The overall proportion of cells infected was relatively equal between conditions, being reduced for all Mce expressing *E. coli* from 2 hpi (Fig. 6f) to 6 hpi (Fig. 6l).

### Invasion of bovine enteroids cells by recombinant *E. coli* expressing Mce protein

**Figure 7:**
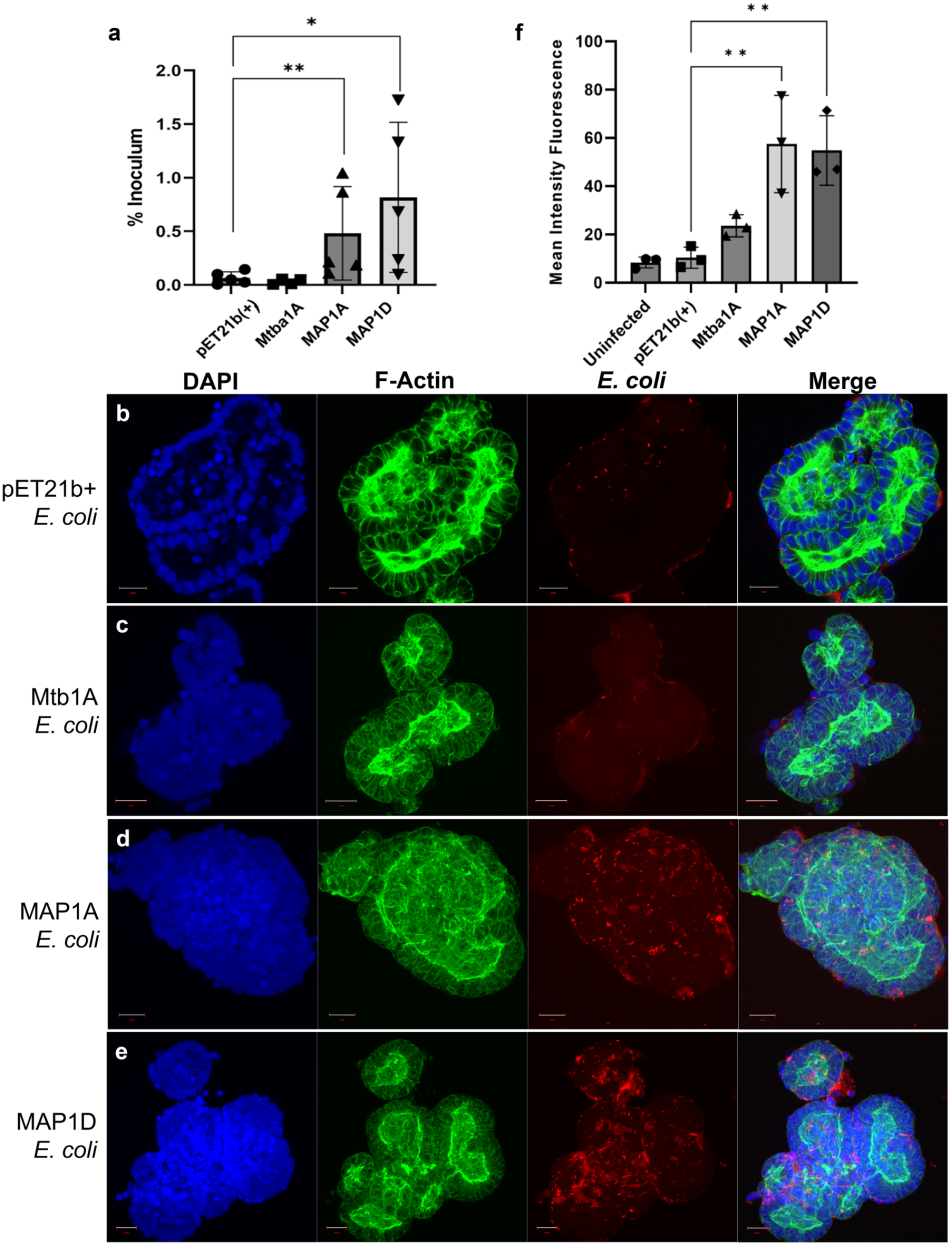
Infection of 3D basal-out bovine enteroids by *E. coli* recombinants. Mce protein expression was induced in *E. coli* recombinants and used to infect 3D basal-out bovine enteroids at an MOI 20 for 2 h. (**a**) % inoculum was calculated from the enteroid lysate CFU/well 2 hpi. Data is representative of the mean of 5 biological replicates from enteroids derived from 2 separate calves ± SD. Statistical analysis performed as a one-tailed student’s T-test compared to the empty vector control. *P* < 0.05 = *; *P* < 0.01 = **. (**b**–**e**) Infected enteroid samples were fixed and stained for nuclei (DAPI, blue), F-actin (Phalloidin, green) and anti-*E. coli* antibody (red). Figures are representative of each infection condition and are maximum intensity projections from Z-stacks. Scale bar = 20 µm. (**f**) Graph depicts quantification of mean intensity fluorescence of the *E. coli* staining normalised against the mean intensity fluorescence of F-actin staining using Fiji software. Data representative of the mean from 3 independent enteroid images per condition ± SD. Statistical analysis performed as a One-way ANOVA and post Hoc Dunnett’s test against the empty vector control (pET21b(+)). *P* < 0.05 = *; *P* < 0.01 = **.

To further investigate the roles of Map1A and Map1D in the attachment of MAP to intestinal epithelial cells, the invasion assays were performed in 3D basal-out bovine enteroids. These enteroids acted as a more physiologically representative model of the lumenal surface of the bovine intestine due to the presence of multiple intestinal epithelial cell types^22^. Following their preparation, the enteroids were sheared to expose the apical surfaces of the epithelial cells and subsequently incubated in suspension with recombinant *E. coli* expressing Mce proteins. The samples were lysed 2 hpi and bacterial abundance determined (CFU/well). To account for differences in bacterial densities in the inoculant preparations used in each experiment, these data were presented as the percentage bacteria recovered compared to the initial inoculum (Fig. 7a).

A significantly greater percentage of bacteria were detected in the enteroids with 2 h of exposure to *E. coli* expressing either Map1A or Map1D compared to enteroids exposed to the pET21b(+) *E. coli* (Fig. 7a). Although the overall level of infection in the enteroids appeared to be low at less than 2% of the original inoculum, this may reflect the lack of phagocytic cells in the enteroid cultures^22^. Expression of Mtb1A, in contrast, did not confer an increased invasive capacity of the *E. coli* into enteroids (Fig. 7a) which differed from data above using MDBK and THP-1 cells (Figs. 3 and 4, respectively).

Confocal microscopy was next used to visualise the infection of the bovine enteroids by the Mce-expressing *E. coli*. Consistent with data above, there was no noticeable attachment of pET21b(+) *E. coli* to the enteroids (Fig. 7b) and little, if any, association of *E. coli* expressing Mtb1A was detected (Fig. 7c). Greater abundance of Map1A and Map1D *E. coli* was present in the samples compared to pET21b(+) *E. coli* (Fig. 7d,e). Quantification of the mean fluorescence intensity (mfi) of the *E. coli* normalised against F-actin staining from these images showed there was significantly greater numbers of Map1A and Map1D *E. coli* compared to the pET21b(+) control (Fig. 7f), corroborating the data shown in Fig. 7a.

## Discussion

Here we show that two *mce* genes encoded by MAP enhance the attachment of *E. coli* to mammalian cells when expressed in the cell membrane. Phagocytic THP-1 cells were used to observe increased attachment and invasion of Mtb1A and Map1A *E. coli*, whereas MDBK cells were used to observe the increased attachment and invasion of Mtb1A and Map1D *E. coli.* Using bovine enteroids as a physiologically representative model of the target host and organ allowed increased attachment and invasion of Map1A and Map1D *E. coli* to be observed, but not Mtb1A *E. coli.* This highlights the importance of using physiologically representative models to fully understand the genes used by MAP in the host-pathogen interaction. The initial interaction between MAP and the host occurs at the intestinal lining, and is a critical step in MAP pathogenesis which may impact the outcome of disease. Therefore, the characterisation of bacterial genes involved in this process deepens our understanding of the initial host-pathogen interaction and may lead to the identification of new therapeutic targets against MAP.

Several studies that have identified potential MAP effector molecules by investigating effects on gene regulation upon exposing the bacteria to the conditions it would experience in a natural infection setting. For example, the exposure of MAP to milk and acidity is considered to upregulate the expression of effector bacterial molecules which aid their attachment to and invasion of host cells^19,20^. From such experiments, FAPs were shown to be upregulated in response to acidic treatment^20^. Although expression of the *mce5* cluster of Mce proteins has been seen to elicit an immune response from infected animals^24^ and be a possible vaccine candidate in goats^25^, and mutations in *mce1D* have been shown to reduce the capacity of MAP to infect MDBK cells^18^, whether the expression of these proteins subsequently aids the attachment and invasion of MAP into intestinal epithelial cells remains to be determined^7^.

In the current study, a role for *mce1A*,* mce1D*,* mce3C* and *mce4A* in the initial interaction between MAP and host cells was investigated. While a role for *mce3C* and *mce4A* could not be elucidated in the current study, this does not definitively mean these genes do not act as effector molecules under different conditions. Previously, *M. bovis* BCG-derived Mce3C was expressed on the surface of *M. smegmatis*, which does not encode its own version of this protein. *M. smegmatis* expressing this protein showed an increased attachment and invasion of murine macrophage-like RAW264.7 cells^17^. In our study, MAP derived *mce3C* was expressed in a Gram-negative *E. coli* host. It is plausible that the outer membrane present in the Gram-negative *E. coli* limits the expression of Mce proteins. For Mce3C and Mce4A, which may require a high level of expression for an observable phenotype, purification of the protein for coating of latex beads may be required to investigate their role in attachment to mammalian cells.

In this study, only the initial interaction of the bacteria with the target cells was studied during the first 6 h of exposure, and this may not be optimal to understand the role of certain Mce proteins in MAP. For example, in other Mycobacteria species, expression of Mce4A was found to be upregulated in bacteria during the stationary phase of growth^26^. This stage is likened to that experienced by the bacterium during the later intracellular phases of the infection process which was not investigated in this paper. However, the role of Mce4A expressed by *M. tuberculosis* was determined using an *E. coli* expression host and was observed to confer increased invasion by 4 hpi in HeLa cells and survival in THP-1 cells up to 24 hpi^15^. Therefore, it may be possible that in contrast to *M. tuberculosis*, Mce4A does not aid attachment and invasion of MAP into host cells*.* While previously there was a low level of protein homology (30%) reported between Mce4A derived from *M. tuberculosis* or *M. avium*^27^, from our analyses there was very high homology (85.22%) (Supplementary Figure S9). However, there was a 21 amino acid deletion at the N-terminus of the MAP Mce4A. While it is unknown if the N terminus is cytoplasmic or extracellular for Mce proteins, if it is extracellular truncation of this part of the protein may limit its ability to interact with eukaryotic cells^16^. This may explain the inability of MAP4A to aid attachment or invasion of eukaryotic cells, whilst maintaining its localisation to the bacterial membrane.

Consistent with other effector molecules identified in MAP, the expression of both *mce1A* and *mce1D* were upregulated upon exposure to acidic conditions^19^. Furthermore, three separate cell culture models were used to investigate the potential effect that the expression that each of these Mce proteins would confer to the attached of *E. coli* to host cells. This suggests that Mce1A enhances attachment and invasion of THP-1 cells, whereas Mce1D enhances attachment and invasion of MDBK cells. It is worth noting that THP-1 cells are phagocytic, whereas MDBK cells are not. Thus, it is plausible that that the mechanisms utilised by each protein to aid infection are different to enable MAP to target distinct cell types in the host.

Morphological changes (altered F-actin staining) were noted in MDBK cells infected with *E. coli* expressing Mtb1A. It is possible that expression of this Mce protein results actin rearrangement to aid bacterial entry into the cell which has been noted for other Mycobacterial effector proteins, including Mce proteins^17^. This change in F-actin morphology was absent in cells infected with *E. coli* expressing MAP-derived Mce proteins, including Mce1D which significantly increased bacterial invasion (Figs. 3a and 5f) in these cells. It is therefore unclear if actin rearrangement is a method utilised in the same way as Mce proteins derived from MAP, or whether MAP possesses other mechanisms for cell entry that were not investigated in this study.

Our data showed that expression of *mce1A* and *mce1D* in *E. coli* enhanced their ability to infect 3D bovine enteroids, whereas expression of *Mtb1A* did not. This indicates these genes exert a degree of host and cell type specificity, and suggests that both the target cell types and receptors for the MAP-derived Mce1A and Mce1D derived proteins are present in the bovine enteroids. This lends credence to the hypothesis that these genes are involved in the initial interaction between MAP and the host at the intestinal lining, and identifies a novel in vitro system in which to study the interactions between MAP and the bovine intestinal epithelium at the molecular level. Our study highlighted that data obtained on the role of specific Mce proteins differed depending on whether cell lines or bovine enteroids were used as sources of host target cells. This clearly demonstrates that physiologically relevant model systems such as enteroid cultures derived from the natural host species should be used in similar in vitro studies aimed at investigating function of MAP effector genes, as cell lines may not reflect the complexity of the in vivo host environment.

## Materials and methods

### Bacterial isolates and culture conditions

DH5α (Invitrogen) and Rosetta 2 (DE3) (Novagen) *E. coli* were cultured in LB broth containing 100 µg/mL ampicillin and 100 µg/mL ampicillin with 34 µg/mL chloramphenicol respectively at 37 °C 180 rpm. The MAP K10 reference strain and MAP C49^28^, a recently isolated field sample, were cultured in 7H9 medium supplemented with 10% Oleic Albumin Dextrose Catalase (OADC) (BD Biosciences), 0.11% glycerol (Sigma Aldrich), 0.1% Tween-80 (Sigma Aldrich), and 1.1 µg/mL Mycobactin J (IDvet) at 37 °C 100 rpm.

### MAP exposure to acid

Five mL of MAP K10 and MAP C49 in the log phase were transferred to 50 mL falcon tubes and centrifuged at 3220 × *g* for 10 min. The cultures were re-suspended in either 5 mL standard 7H9 medium or 5 mL of 7H9 medium made to pH 3.0 prior to being autoclaved. Cultures were then immediately centrifuged at 3220 × *g* for 10 min for RNA isolation to serve as a control, or cultured at 37 °C 100 rpm for 2 h. The cultures were then centrifuged at 3220 × *g* for 10 min for RNA isolation. All experiments were carried out in three biological replicates.

### RNA isolation from MAP

Immediately after pelleting, bacterial pellets were re-suspended in 1 mL Trizol reagent (Thermo Fisher Scientific) and homogenised using lysing matrix B beads (MP Biomedicals). Tubes were pulsed in a Fastprep machine at 6.0 speed for 30 s twice followed by 6.5 speed for 45 s, keeping samples on ice for 5 min between steps. The sample was centrifuged at 16,200 × *g* for 3 min and the supernatant transferred into a screw-top micro-centrifuge tube. 200 μL chloroform was added to the suspension and the sample treated for RNA isolation as described by the manufacturer, with some adjustments. Namely, after centrifugation with isopropanol only 50% of the supernatant was removed, and all centrifugation steps were performed at 12,000 × *g* for 20 min.

### RT-qPCR

cDNA was synthesised from the RNA using Agilent AffinityScript Multiple Temperature cDNA Synthesis Kit according to the manufacturer’s instructions. Non-reverse transcriptase controls were used to confirm the absence of genomic DNA from the samples.

All qPCR experiments were performed using PerfeCTa SYBR green Supermix (Quantabio, VWR international Ltd). The total reaction volume was 10 µL consisting of 5 µL Supermix, 0.5 µL 10 µM forward and reverse primers (Table 1) at pre-determined optimal concentrations, 1.5 µL nuclease free water and 2.5 µL template. Samples were loaded in triplicate into 96 well plates and non-template controls were included to verify the absence of contamination. Oligonucleotides were designed using Primer3^29,30^ and Netprimer (Biosoft International) software and are outlined in Table 1 using the “conventional” PCR primers to generate PCR amplicons to act as the template for individual standard curves. The experimental cDNA was diluted 1:20 to generate template for the RT-qPCR reaction. The relative quantities of mRNA were calculated using the Pfaffl method^31^, using the geometric mean of the RT-qPCR results for the reference genes *GAPDH* and *1g2*^32,33^ to calculate differences in the template RNA levels for standardisation of the Ct values for the genes of interest.

**Table 1:** Primer pairs used to amplify and then quantify specified genes using qPCR.

Gene	Primers (5’-3’)	Product length (bp)	Melting Temp (°C)
qPCR			
MAP mce1A For	GTCACCGCAGAAGATCACCC	184	60.74
MAP mce1A Rev	CACTGACTGGCCGAACTTCT		59.97
Conventional			
MAP mce1A For	TCAAGCTGATCCCGTCGAAC	517	60.11
MAP mce1A Rev	CCGCCCTTGTTGAAGAGGTC		60.69
qPCR			
MAP mce1D For	CGCAACAGCATCACCAACAT	128	59.76
MAP mce1D Rev	GTCGTGTTGAACTGCTTGCC		60.32
Conventional			
MAP mce1D For	ACCAGAACAAGTACCGGGTG	581	59.6
MAP mce1D Rev	CTCCAGGTTCTTGACGTCGT		59.69
qPCR			
MAP mce3C For	CTGCTGGACGAACGGGATT	124	65.2
MAP mce3C Rev	TTTGAGCTGGGTTCGGTTGT		65.4
Conventional			
MAP mce3C For	CGATCTGACCACCACCATCA	576	64.7
MAP mce3C Rev	TCCAGGAAACGGTCGTACAT		64
qPCR			
MAP mce4A For	AACCTGCCCACGATCAACAA	133	59.97
MAP mce4A Rev	TGGTGTCGATGAAGTCCTGC		60.11
Conventional			
MAP mce4A For	ATCGACCTGCTGCACAAGAT	542	60.18
MAP mce4A Rev	TCGGGATAGGTGTACGACGG		60.67
qPCR			
MAP gapDH For	CTACACCCAGGACCAGAACC	134	59.39
MAP gapDH Rev	CCTTGAGGTTGGGCATGAC		58.43
Conventional			
MAP gapDH For	CGGCAGCCAGAACATCATCT	469	60.46
MAP gapDH Rev	GGCTTGGTTGTCGATCACCT		60.32
qPCR			
MAP 1g2 For	GCTTCGCGATACTTCCAACG	119	64.3
MAP 1g2 Rev	CGCGTCACCGGACCAG		65.3

### Cloning, expression and expression of Mce1A, Mce1D, Mce3C and Mce4A

Genomic DNA from *Mycobacterium tuberculosis* H37Rv (ATCC 25618) was obtained from Dr. Robin Skuce (Agric-Food & Biosciences Institute), and genomic DNA from MAP K10 was extracted using the Qiagen Blood and Tissue kit. Briefly, the bacterial pellet was re-suspended in enzymatic lysis buffer (20 mM Tris-Cl pH 8.0, 2 mM sodium EDTA, 1.2% Triton X-100) containing 40 mg/mL lysozyme and incubated at 37 °C for 16 h. Genomic DNA extraction was then performed according to the manufacturer’s instructions.

Full length *mce1A* from *M. tuberculosis* (Rv0169), *mce1A* from MAP K10 (MAP3604), *mce1D* from MAP K10 (MAP3607), *mce3C* from MAP K10 (MAP2114c) and *mce4A* from MAP K10 (MAP0564) genes were amplified from genomic DNA by PCR using the primers listed in Table 2. These genes will be referred to as *mtb1A*, *map1A*, *map1D*,* map3C* and *map4A* respectively henceforth. Each forward primer contained an *Nde*I restriction enzyme site and each reverse primer contained an *Xba*I restriction site.

**Table 2:** Primer pairs used to amplify the appropriate mce gene for subsequent cloning steps.

Genome region	Primer	Sequence (5'-3)	Product size (bp)
mce1A (*M. tuberculosis*)	Mtb1A For	ATATAT **TCTAGA** AAGGAGAAATAATATACGACGCCGGGGAAG	1365
	Mtb1A Rev	ATATAT **CATATG** TGGGTTGATCGTGTTATC	
mce1A (MAP)	MAP1A For	ATATAT **TCTAGA** AAGGAGAAATAATATGCCCGACCCGTCCAG	1302
	MAP1A Rev	ATATAT **CATATG** TGGGTTGATCGTGTTATC	
mce1D (MAP)	MAP1D For	ATATAT **TCTAGA** AAGGAGAAATAATATAGCACCATCTTTGA	1611
	MAP1D Rev	ATATAT **CATATG** CTGGCCACCTCCGAAG	
mce3C (MAP)	MAP3C For	ATATAT **TCTAGA** AAGGAGAAATAATATACGTGGAAGCTACC	1176
	MAP3C Rev	ATATAT **CATATG** TGGCTGATCGAATTC	
mce4A (MAP)	MAP4A For	ATATAT **TCTAGA** AAGGAGAAATAATATGTGGCTTTCGCTG	1137
	MAP4A Rev	ATATAT **CATATG** GAAGTCGTCCCGTTC	

Restriction enzyme sites are in bold.

PCRs were performed in 25–50 µL volumes using 10 ng template DNA, 1 µM oligonucleotide primers, 200 µM deoxynucleotides triphosphate, 0.04 U/mL Phusion High Fidelity Polymerase (Thermo Fisher Scientific), 1 × GC buffer, 6–10% DMSO and 5 mM MgCl_2_ depending on the primer pair used. 35 cycles of DNA amplification were performed. The samples were denatured at 98 °C for c. 5 min, annealed at c. 52 °C and extension at 72 °C (allowing 30 s per 500 bp of DNA to be synthesised). The amplicons were cloned into pET21b(+) to generate the appropriate *mce* construct with a hexa histidine tag. Both DH5α and Rosetta 2 (DE3) strains of *E. coli* were transformed with the recombinant plasmids for expression studies. The presence of the inserts was confirmed by PCR, restriction enzyme digestion and sequencing.

Overnight cultures of *E. coli* Rosetta 2 (BL-21) (Novagen) containing the recombinant expression plasmids was diluted 1:10 in fresh LB broth containing 100 µg/mL ampicillin and 35 µg/mL chloramphenicol and cultured at 37 °C 180 rpm to an optical density (OD) of 0.6 at 600 nm. For transcription induction, IPTG (isopropyl thio-b-D-galactoside, Sigma) to a final concentration of 0.1 mM was added to the culture and incubation was continued for 2 h.

### Subcellular fractionation

Recombinant *E. coli* were cultured in 200 mL LB broth containing 100 µg/mL ampicillin and 35 µg/mL chloramphenicol at 37 °C 180 rpm to an OD of 0.6 at 600 nm and then Mce protein expression was induced with 0.1 mM IPTG for a further 2 h. Cells were harvested by centrifugation at 3000 × *g* for 10 min. The membranous fractions were prepared as described in Schell et al.^34^.

Briefly, The pellet was washed with 0.1 volume of TM buffer (20 mM Tris-HCl pH 7.0, 3 mM MgCl_2_) and re-pelleted at 3000 × *g* for 10 min and frozen at − 80 °C. The pellet was then suspended in 3 mL of 10 mM Tris-HCl pH 7.0, 25% sucrose (w/v). A cocktail of protease inhibitors was added at 5 mL per gram of wet pellet and lysozyme was added to 0.5 mg/mL (w/v) and incubated at 37 °C for 20 min. MgCl_2_ was then added to a final concentration of 3 mM and incubated at 37 °C for 20 min. One volume of 4% Triton-X100 was added and mixed for 4 min before freezing the solution at − 80 °C and subsequently thawed at 37 °C and mixed for 1 min. This freeze-thaw cycle was repeated for a second time before the supernatant was removed after centrifuging the sample at 7500 × *g* for 15 min and frozen at − 80 °C. The supernatant was thawed at room temperature and ultra-centrifuged at 110,000 × *g* for 1 h at 5 °C to pellet the crude outer membranes.

The supernatant was stored and represents the cytoplasmic fraction of the bacteria; the pellet was resuspended in 0.3 mL TM by bath sonification for 5 min. One volume of 4% Triton-X100 was added and incubated on ice for 30 min before centrifuging at 7500 × *g* for 15 min. The supernatant was aliquoted into a separate tube and ultra-centrifuged at 110,000 × *g* for 1 h. The pelleted membranes were suspended in 0.4 mL TM by sonification and centrifuged at 7500 × *g* for 15 min. The supernatant containing the outer-membranes of the bacteria were removed and diluted fourfold with 5 mM Tris-HCl pH 8.0. The outer membranes were pelleted by ultracentrifugation at 110,000 × *g* for 1 h. The pellet was suspended using sonification in 0.6 mL 5 mM Tris-HCl pH 8.0 and RNase was added to 0.5 mg/mL (w/v). This was incubated at 37 °C for 10 min and EDTA was added to 10 mM and incubated for a further 40 min. The sample was then adjusted to 50 mM Na_2_CO_3_ and 1 M NaCl and incubate on ice for 1 h before incubation at 37 °C for 15 min. The membranes were centrifuged at 110,000 × *g* for 1.25 h and washed by sonification in 0.1 mL 100 mM Na_2_CO_3_ and 1 M NaCl. The sample was incubated on ice for 30 min and pelleted at 110,000 × *g* for 1 h. The pellet was washed again by sonification in 0.1 mL 100 mM Na_2_CO_3_ and 1 M NaCl and re-pelleted as described.

### Detection of protein expression using Western Blotting

The final fractions representing the cytoplasm and membrane, cytoplasm alone, and two membrane fractions at different wash periods were selected to analyse by western blot to detect the presence of the His-tagged Mce proteins in the membrane and DNAK in the cytoplasm of the bacteria.

To this end, samples were denatured in sample treatment buffer (50 mM Tris-HCl pH 6.8, 4% SDS (w/v), 20% sucrose (w/v), 0.0001% bromophenol blue) containing 2% β-mercapto-ethanol at a 1:1 ratio. The samples were boiled at 100 °C for 10 min immediately prior to protein separation by SDS-PAGE. Transfer of the protein to a nitrocellulose membrane was performed using an iBlot2 Gel Transfer Device (Life Technologies). Membranes were blocked using PBS-T (phosphate buffered saline, 0.1% Tween-20 (v/v)) containing 5% non-fat milk protein for 1 h at room temperature. Membranes were incubated with the primary antibody prior to being washed three times in PBS-T followed by incubation with the secondary antibody. Protein separation was visualised using Licor software.

To detect the C-terminal His-tagged recombinant Mce protein in the *E. coli*, anti-His monoclonal antibody (Raybiotech) and goat anti-rabbit IgG were used as primary and secondary antibodies (Supplementary Table S1).

### Cell culture and invasion assays

MDBK cells (gifted by Dr. Spring Tan) were cultured in DMEM w. 100 U/mL Pen/Strep/Glutamine (Gibco), 1X non-essential amino-acids (Gibco), 5% heat-inactivated horse-serum (Sigma) and 1 mM sodium pyruvate (Gibco).

THP-1 cells (gifted by Prof. David Hume’s lab) were cultured in suspension RPMI w. 1X Glutamax (Gibco) and 10% heat inactivated foetal bovine serum (v/v) (Gibco) at 37 °C and 5% CO_2_. For THP-1 differentiation into macrophages, cells were seeded in 24 well tissue plates at a density of 2.5 × 10^5^ cells/well in the presence of 200 nM phorbol 12-myristate 13-acetate (PMA) (VWR, UK) for three days, before being left to rest for 2 days in RPMI culture media.

Invasion of MDBK cells seeded at 1 × 10^5^ cells per well into a 24-well plate by Rosetta 2 (BL-21) transformed with pET21b(+)/*mtb1A*, pET21b(+)/*map1A*, pET21b(+)/*map1D*, pET21b(+)/*map3C*, pET21b(+)/*map4A* and empty vector pET21b(+) was assayed. *E. coli* were cultured overnight and diluted 1:10 in fresh medium containing 100 µg/mL ampicillin and 35 µg/mL chloramphenicol and incubated at 37 °C 180 rpm to reach an OD of 0.6 at 600 nm and induced with 0.1 mM IPTG for 2 h at 37 °C. Following induction, the bacteria was normalised to an OD of 0.6 at 600 nm and centrifuged at 3000 × *g* for 10 min and re-suspended in mammalian cell culture medium. Prior to the infection, fresh medium replaced the mammalian cell culture medium. Recombinant *E. coli* cells were added to the monolayer at a multiplicity of infection (MOI) of 20:1 for MDBK cells and an MOI of 10:1 for THP-1 cells, which had undergone differentiation to macrophages as described, and incubated at 37 °C for 1 h. The cells were washed three times with PBS, and fresh cell culture medium was added to the infected cells for a further 1 or 5 h for incubation at 37 °C. For THP-1 invasion assays, the fresh medium contained 10 µg/mL gentamicin to kill extracellular bacteria.

### Enteroid generation and infection

3D basal-out bovine enteroids were isolated from the small intestine and maintained as described in^22,23^. Briefly, enteroids were suspended in Matrigel domes and cultured with murine IntestiCult (STEMCELL) supplemented with 10 µM Y-27632, 10 µM LY2157299 and 55 nM SB202190 (henceforth referred to as complete IntestiCult medium). Medium was changed every 2–3 days and were passaged after 7–10 days of culture. To passage, the medium was removed and the Matrigel containing enteroids was suspended in 1 mL of ice-cold DMEM/F12. The wells of enteroids were pooled and centrifuged at 400 × *g* for 5 min. All bar 1 mL supernatant was removed, into which the enteroids were re-suspended and sheared by mechanical pipetting. The sheared enteroids were counted using a Brightfield microscope, and the fragments were suspended in Matrigel so that 200 fragmented enteroids were present in 50 µL of Matrigel per well. 650 µL fresh complete IntestiCult medium was used to culture the enteroids.

The enteroids were infected as described in^23^. Briefly, the enteroids were pooled and sheared as described above. The number of enteroid fragments were counted for 2000 fragments to be seeded per well. Each fragmented enteroid contained on average 100 cells based on previous gDNA quantification per well^23^. The fragment was suspended in the appropriate volume of inoculum for each recombinant *E. coli* strain so that there was a final MOI of 20 per bovine cell to recapitulate what was observed in the MDBK epithelial cell line.

The recombinant *E. coli* strains were prepared for infection as described previously, and the sheared enteroids were exposed to the *E. coli* inoculum in a suspension of complete IntestiCult medium. The enteroid and bacteria suspension were incubated at 37 °C for 2 h, pelleted at 500 rpm and washed with PBS. Enteroids were sheared open by mechanical pipetting to release any *E. coli* trapped in the enteroid lumen that remained unattached from the cell surface. The enteroids were washed three more times with PBS and lysed using 0.1% Triton X100. The cell lysates were plated onto LB plates containing 100 µg/mL carbenicillin and 35 µg/mL chloramphenicol.

### Confocal microscopy

To image the infection of mammalian cell lines by recombinant *E. coli*, cells were cultured on coverslip slides prior to infection and fixed with 2% paraformaldehyde (PFA) for 20 min at room temperature. For the bovine enteroid samples, the samples were first pelleted at 400 × *g* and re-suspended in 4% PFA (w/v) and fixed for 1 h at 4 °C. Samples to be stained were then permeabilised with 0.1% Triton-X100 (v/v) for 15 min at room temperature and blocked with PBS containing 0.5% bovine serum albumin (v/v) and 0.02% sodium azide (w/v) (termed blocking buffer) for 30 min at room temperature. The samples were incubated with the designated primary antibody diluted to the appropriate concentration in blocking buffer (Supplementary Table 1) for 1 h at room temperature, washed three times with PBS, and incubated with the appropriate secondary antibody diluted in blocking buffer (Supplementary Table 1) for 1 h. Where required, samples were also incubated with phalloidin (ThermoFisher) for 20 min. Samples were then washed three times and nuclei stained with 300 nM DAPI for 2–5 min. The coverslips were mounted on a glass slide using Prolong Gold. Enteroids were adhered to the surface of a glass slide using the CytoSpin method described in^23^. Slides were visualised using Leica LSM710 upright immunofluorescence (IF) microscope and processed using Zen Black software. Where necessary, images were process using Image Studio Lite to change contrast which was applied equally to control images.

Mean fluorescence intensity (mfi) of images was quantified using 3 independent confocal images as described in Shihan et al.^35^. Briefly, images were taken settings on the LSM710 confocal microscope using the same for fluorescence gain, digital offset and digital gain, using a negative control to eliminate background staining of secondary antibody controls. The fluorescence intensity was quantified using Fiji (ImageJ) for each image. The region of interest was selected using the drawing tool, and the fluorescence intensity of the DAPI (blue), F-actin (green) and *E. coli* (red) staining was measured using the ‘split channel’ function. The measurements were also taken for an unstained section of each image to eliminate background mfi from the values. The mfi of *E. coli* was normalised against the mfi of F-actin to account for variation in enteroid size and number of cells present between each sample.

### Statistical analysis

Results are expressed as the mean of biological replicates ± standard deviation (SD). Statistical analysis was performed in GraphPad Prism using a one-way ANOVA followed by a post hoc Dunnett’s test for statistical significance.

### Supplementary Information

Supplementary Information.

## Data Availability

The data that support the findings of this study are available from Rosemary Blake upon request.
