# Supplementary material for: The role of Mce proteins in Mycobacterium avium paratuberculosis infection
Source: Sci Rep. 2024 Jun 28;14:14964. doi: 10.1038/s41598-024-65592-2 (PMC11213854; doi:10.1038/s41598-024-65592-2)
Supplement: Supplementary file 1 — Supplementary Information. [file 41598_2024_65592_MOESM1_ESM.pdf]

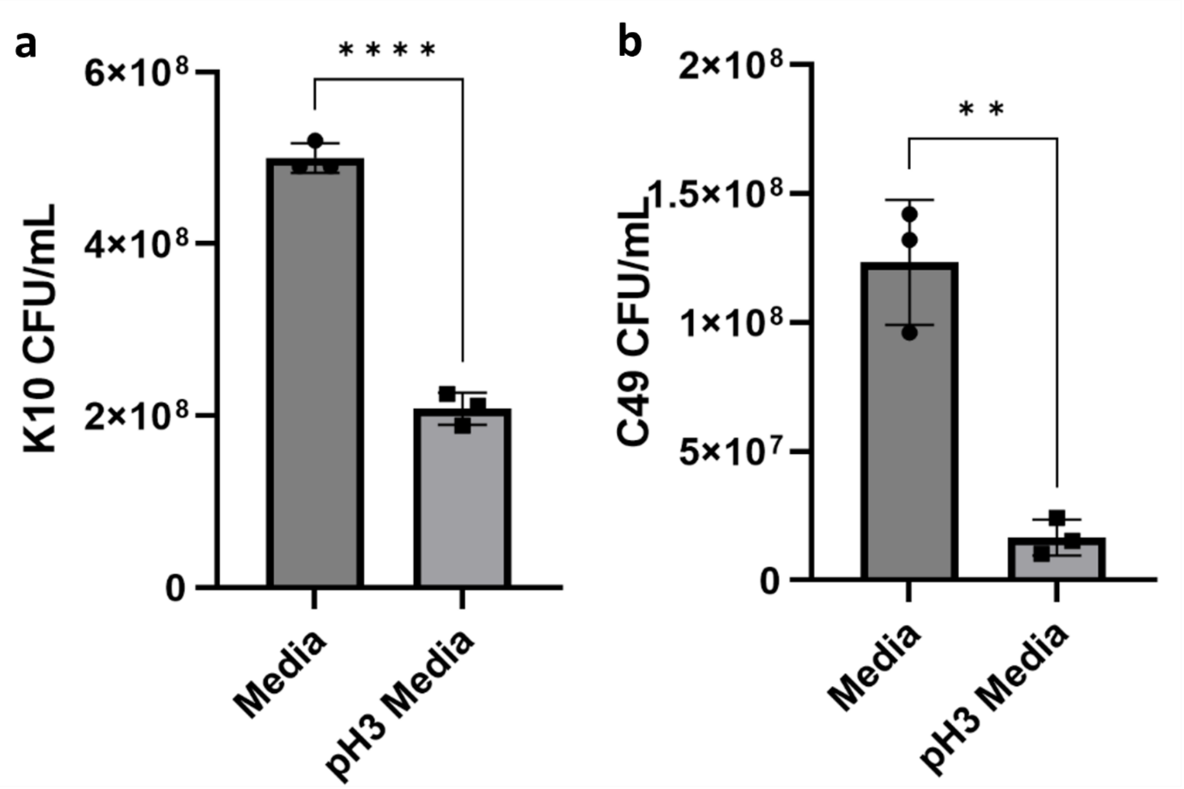

Supplementary Figure S1| CFU/ml values of MAP cultured in acidic 7H9 medium. MAP was cultured to an OD_{600} 0.6 and pelleted. The pellet was re-suspended in 7H9 growth media that was either the standard pH or pH 3.0 and cultured at 37°C 100 rpm for 2 hours. The cultures were then diluted and plated onto 7H10 agar and incubated at 37°C for up to 6 weeks. A) MAP K10 CFU values; B) MAP C49 CFU values. Data analysed using Student's unpaired T-test. P<0.01 = **; P<0.0001 = ****.

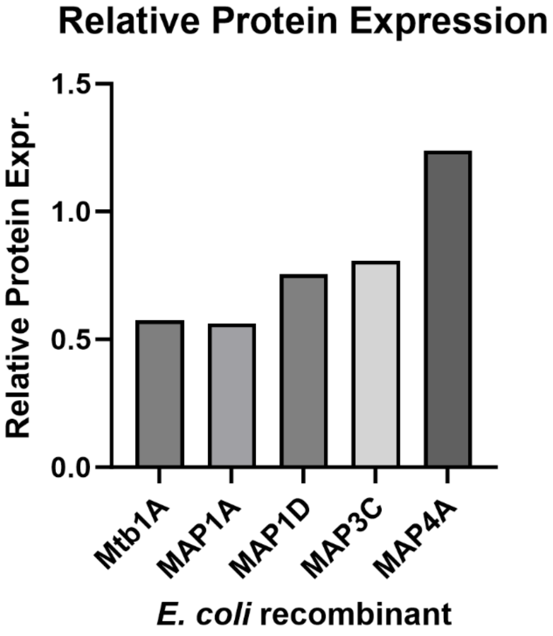

Supplementary Figure S2| Relative protein expression of MAP derived Mce proteins expressed in the recombinant *E. coli* membrane. Relative protein expression of His-tagged Mce protein in membrane fraction 1 was normalised against DNAK protein expression in the membrane & cytoplasm fractions of the Western Blots shown in Figure 2. ImageJ was used to quantify pixel intensity of both the DNAK control and the Mce protein and the relative protein expression is shown as a ratio of Mce/DNAK.

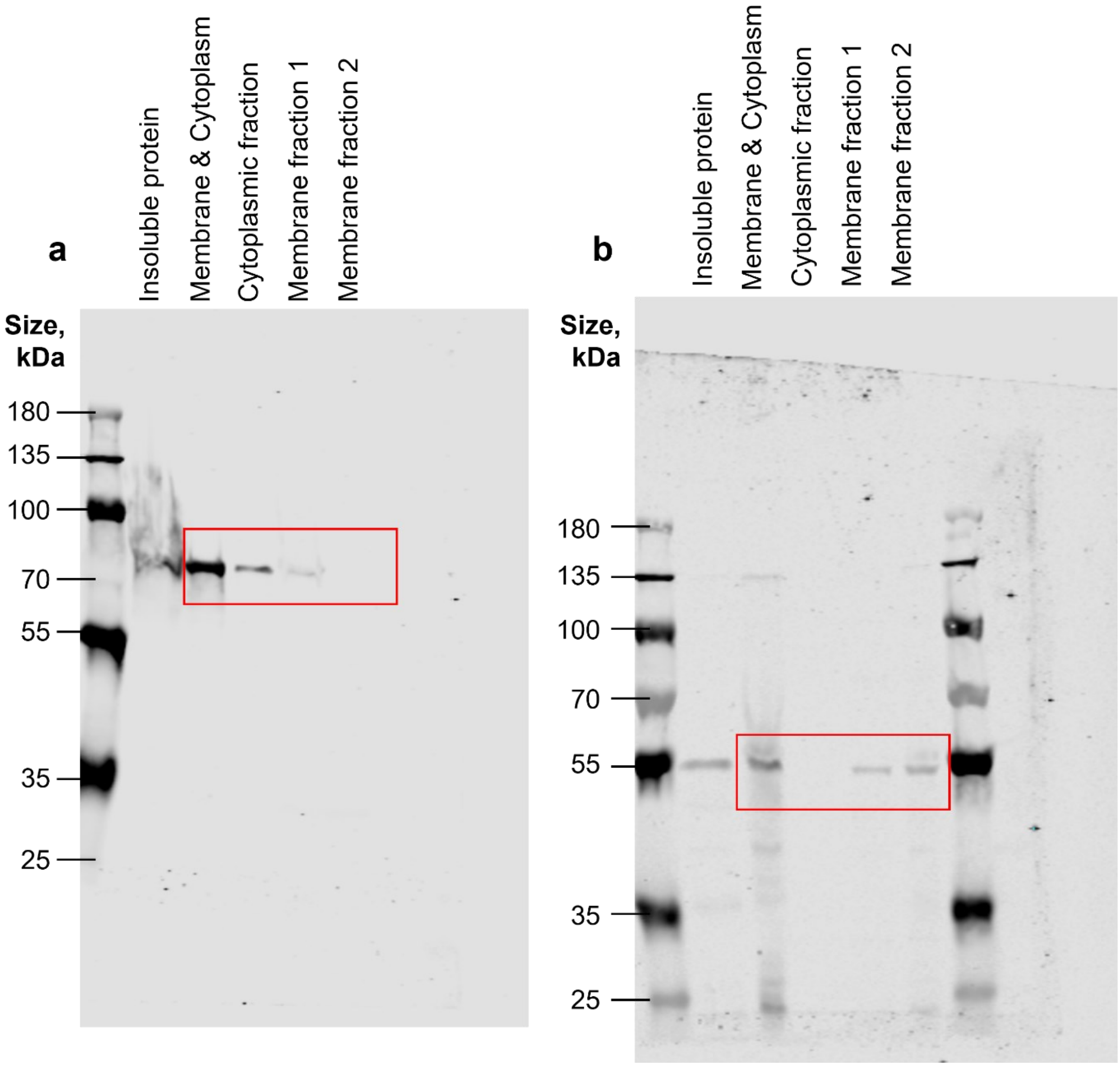

Supplementary Figure S3| Subcellular fractionation of *E. coli* recombinant expressing Mce1A derived from *M. tuberculosis.* *Mce protein expression was induced with 0.1 mM IPTG for 2 hours at 37°C 180rpm. The bacteria were then separated into fractions of the insoluble protein, the cell membrane and cytoplasm, the cytoplasm alone and two separate washes of the cell membrane. The fractions were separated by SDS-PAGE and electro-transferred to a nitrocellulose membrane.* ***a)*** *Rabbit monoclonal anti-DNAK antibody was used as an E. coli cytoplasmic control (70 kDa).* ***b)*** *Rabbit monoclonal anti-His antibody was used to detect the His-tagged Mce protein to determine its location in the bacteria. Red box indicates the cropped images presented in Figure 2.*

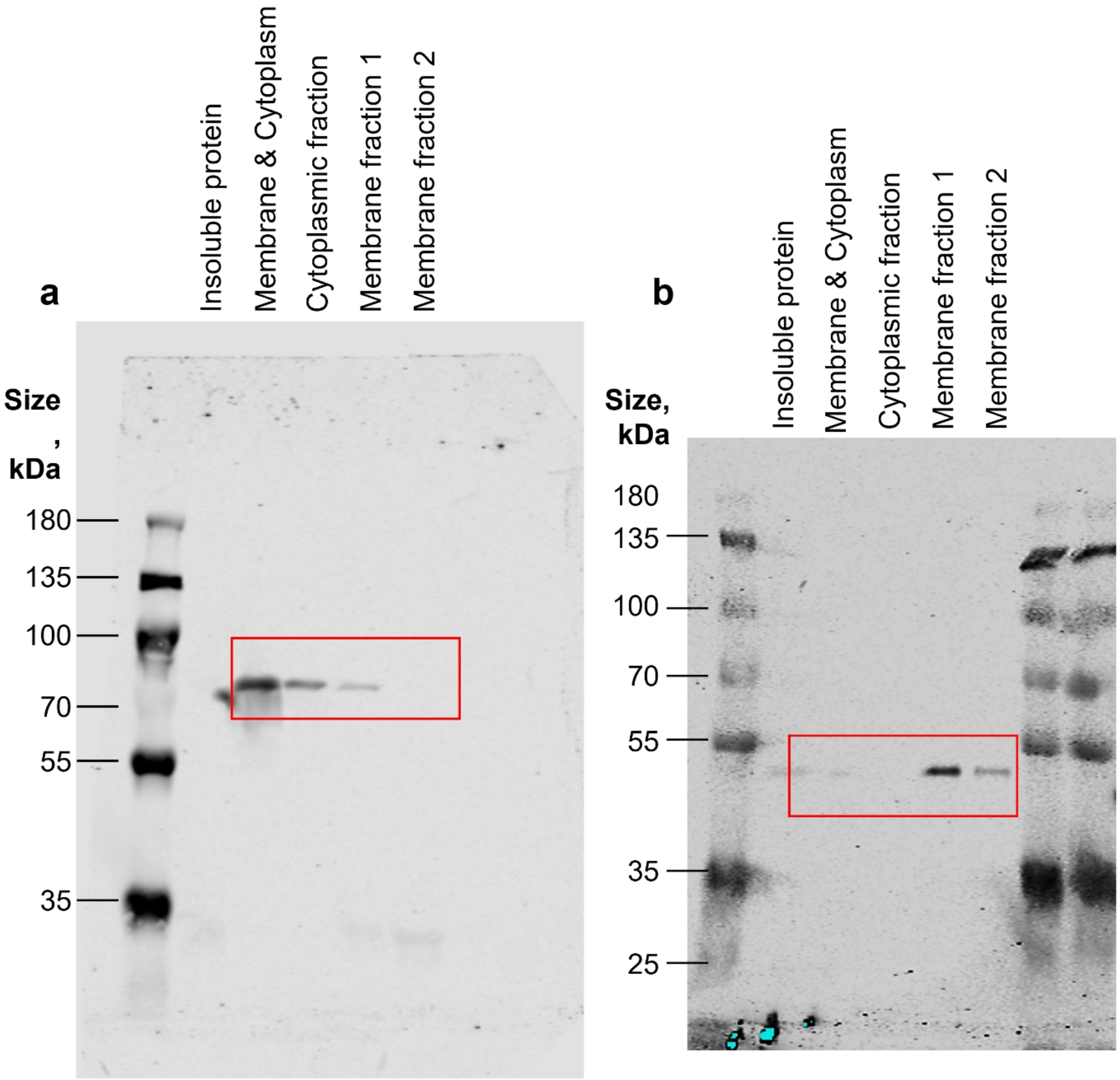

Supplementary Figure S4| Subcellular fractionation of *E. coli* recombinant expressing Mce1A derived from MAP*. Mce protein expression was induced with 0.1 mM IPTG for 2 hours at 37°C 180rpm. The bacteria were then separated into fractions of the insoluble protein, the cell membrane and cytoplasm, the cytoplasm alone and two separate washes of the cell membrane. The fractions were separated by SDS-PAGE and electro-transferred to a nitrocellulose membrane.* ***a)*** *Rabbit monoclonal anti-DNAK antibody was used as an E. coli cytoplasmic control (70 kDa).* ***b)*** *Rabbit monoclonal anti-His antibody was used to detect the His-tagged Mce protein to determine its location in the bacteria. Red box indicates the cropped images presented in Figure 2.*

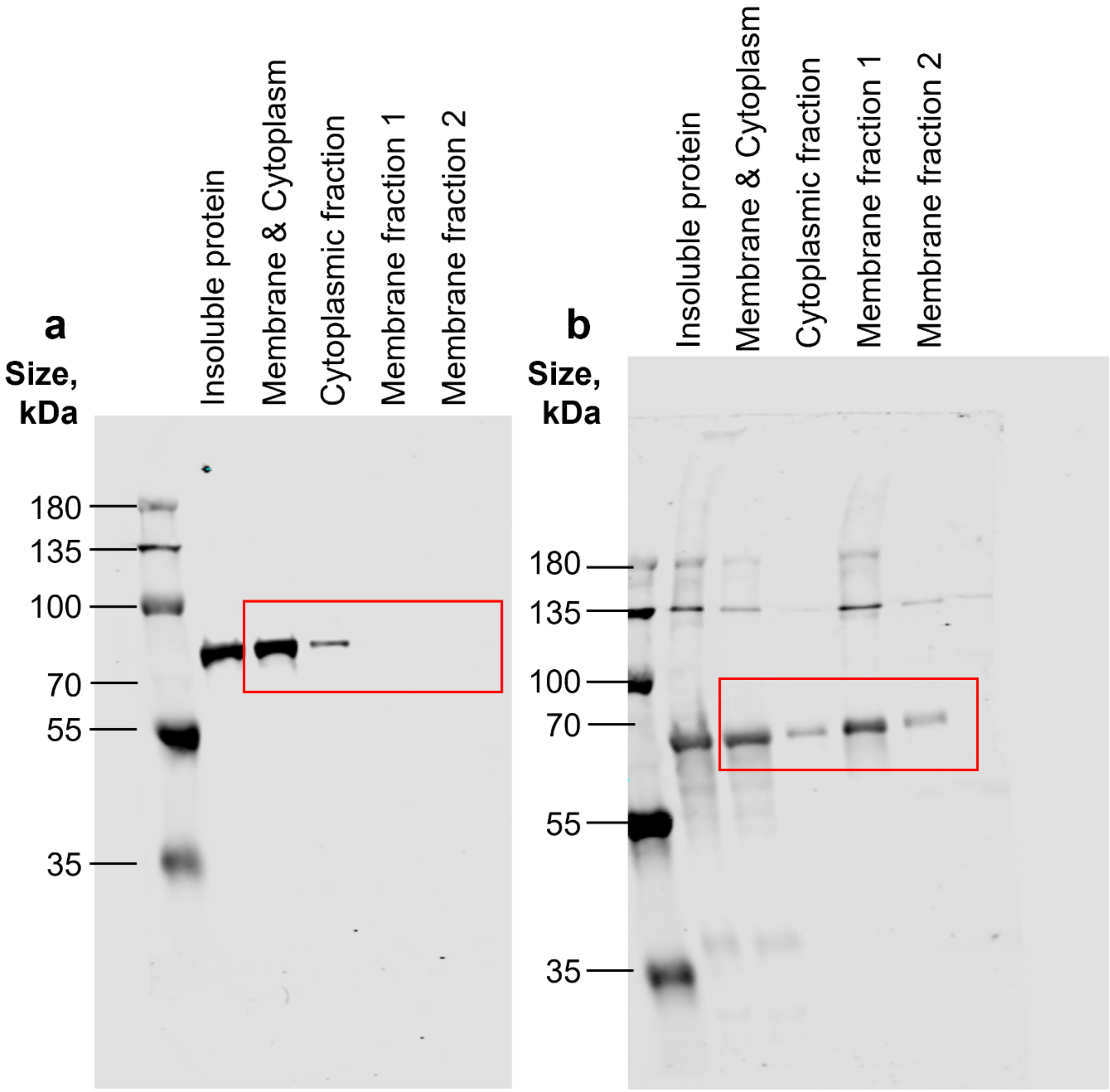

Supplementary Figure S5| Subcellular fractionation of *E. coli* recombinant expressing Mce1D derived from MAP. *Mce protein expression was induced with 0.1 mM IPTG for 2 hours at 37°C 180rpm. The bacteria were then separated into fractions of the insoluble protein, the cell membrane and cytoplasm, the cytoplasm alone and two separate washes of the cell membrane. The fractions were separated by SDS-PAGE and electro-transferred to a nitrocellulose membrane.* ***a)*** *Rabbit monoclonal anti-DNAK antibody was used as an E. coli cytoplasmic control (70 kDa).* ***b)*** *Rabbit monoclonal anti-His antibody was used to detect the His-tagged Mce protein to determine its location in the bacteria. Red box indicates the cropped images presented in Figure 2.*

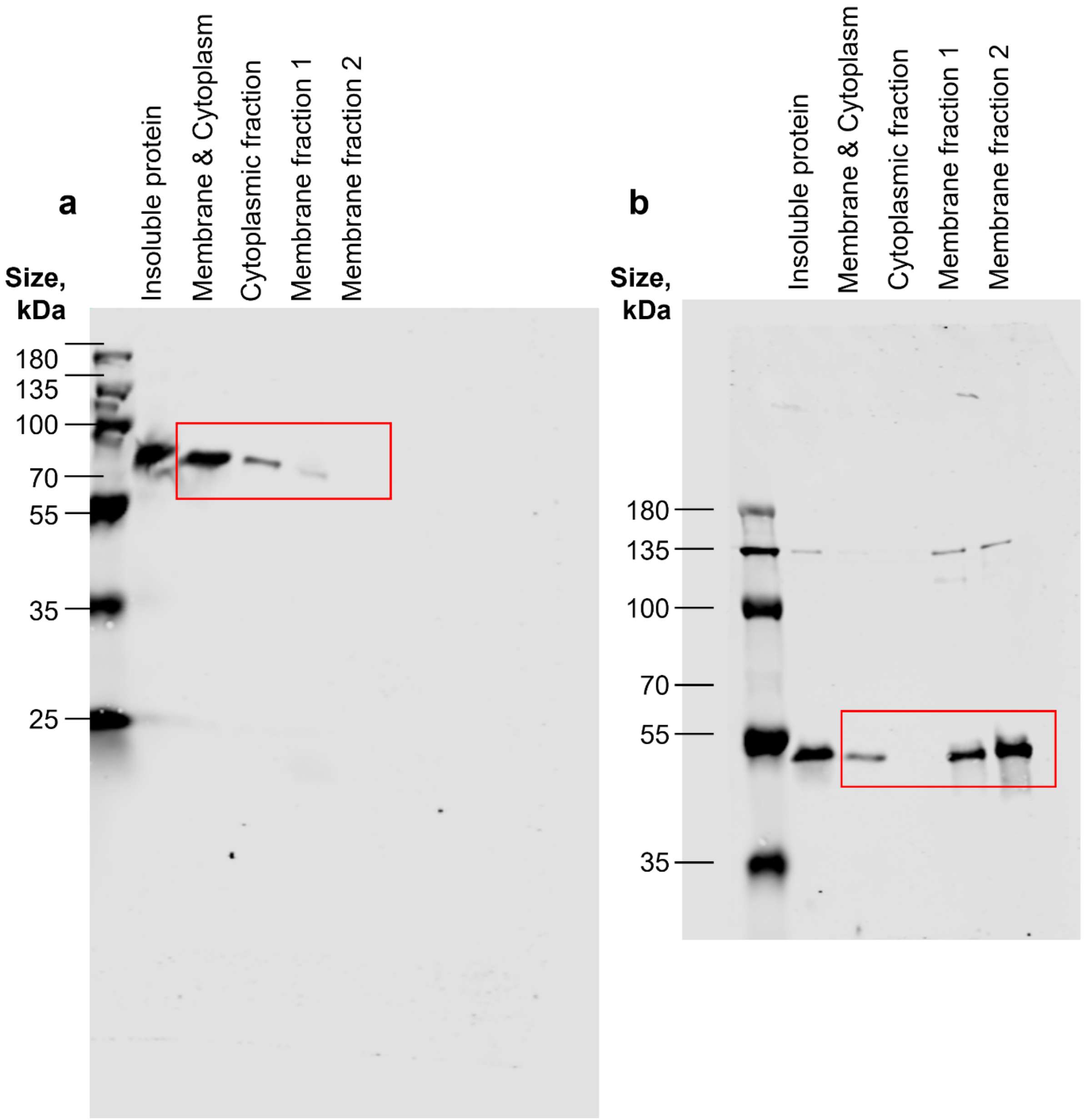

Supplementary Figure S6| Subcellular fractionation of *E. coli* recombinant expressing Mce3C derived from MAP*.* *Mce protein expression was induced with 0.1 mM IPTG for 2 hours at 37°C 180rpm. The bacteria were then separated into fractions of the insoluble protein, the cell membrane and cytoplasm, the cytoplasm alone and two separate washes of the cell membrane. The fractions were separated by SDS-PAGE and electro-transferred to a nitrocellulose membrane.* ***a)*** *Rabbit monoclonal anti-DNAK antibody was used as an E. coli cytoplasmic control (70 kDa).* ***b)*** *Rabbit monoclonal anti-His antibody was used to detect the His-tagged Mce protein to determine its location in the bacteria. Red box indicates the cropped images presented in Figure 2.*

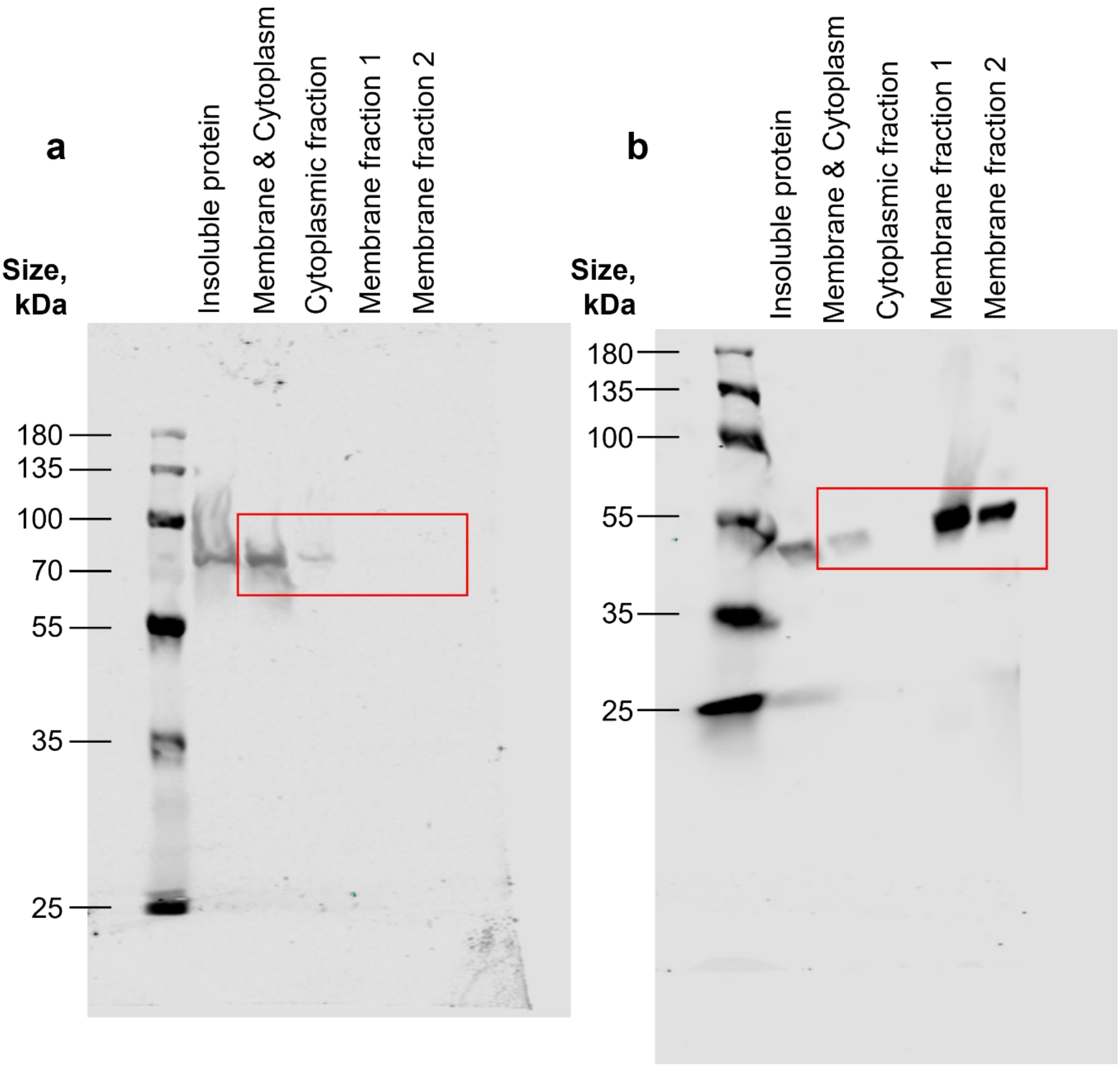

Supplementary Figure S7| Subcellular fractionation of *E. coli* recombinant expressing Mce4A derived from MAP*. Mce protein expression was induced with 0.1 mM IPTG for 2 hours at 37°C 180rpm. The bacteria were then separated into fractions of the insoluble protein, the cell membrane and cytoplasm, the cytoplasm alone and two separate washes of the cell membrane. The fractions were separated by SDS-PAGE and electro-transferred to a nitrocellulose membrane.* ***a)*** *Rabbit monoclonal anti-DNAK antibody was used as an E. coli cytoplasmic control (70 kDa).* ***b)*** *Rabbit monoclonal anti-His antibody was used to detect the His-tagged Mce protein to determine its location in the bacteria. Red box indicates the cropped images presented in Figure 2.*

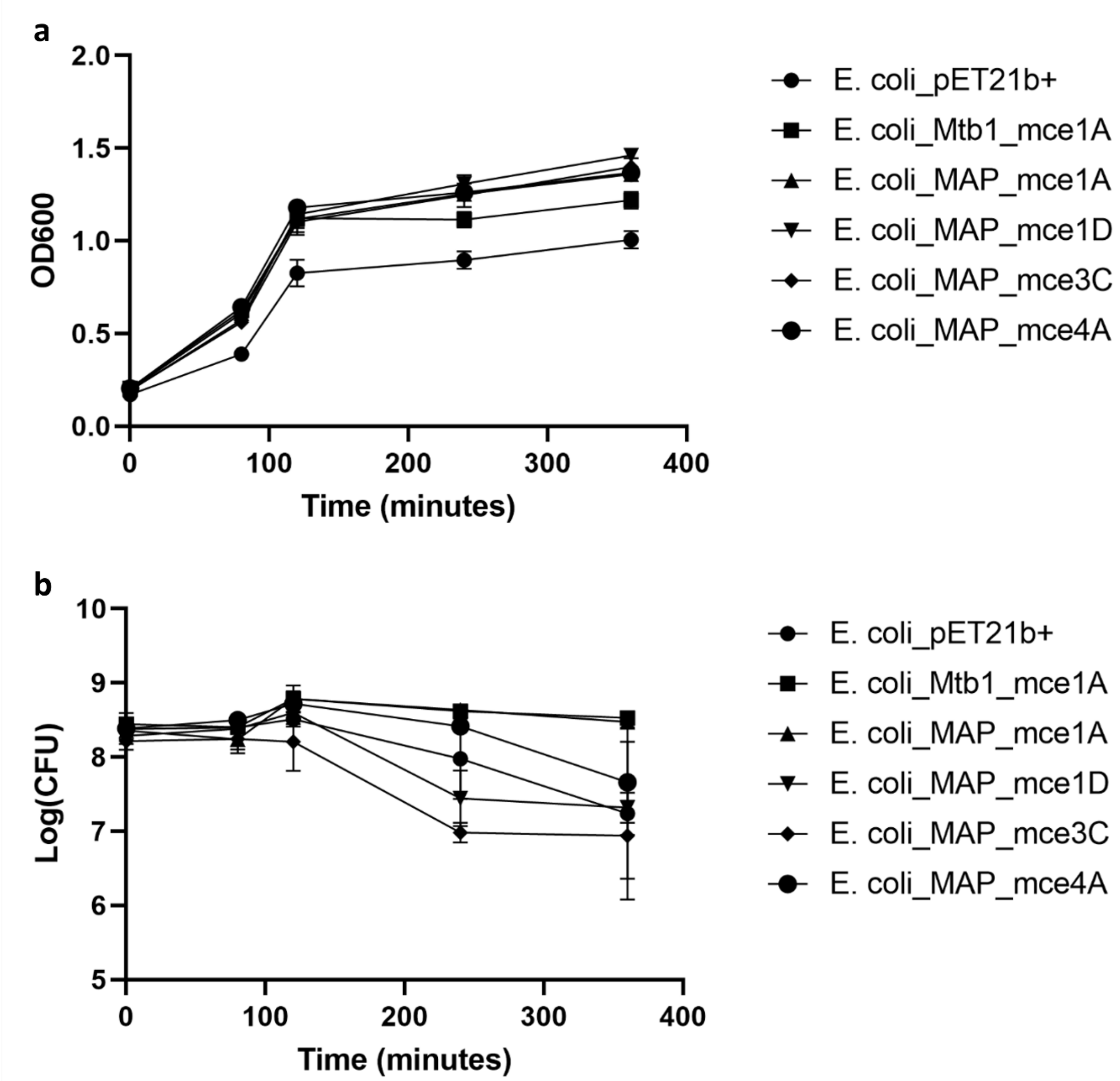

Supplementary Figure S8| Growth curves of recombinant E. coli mutants upon induction of Mce protein expression. 1:10 dilution was performed from an overnight culture of recombinant *E. coli* clones and cultured at 37°C. OD_{600} **a)** and CFU/mL **b)** values were taken at the indicated times, and protein production was induced with 0.1 mM IPTG upon an OD_{600} value of 0.6 being reached. Bacteria were cultured on LB agar containing the relevant antibiotics and cultured overnight at 37°C for CFU/mL analysis. Results were gained from 3 biological replicates.

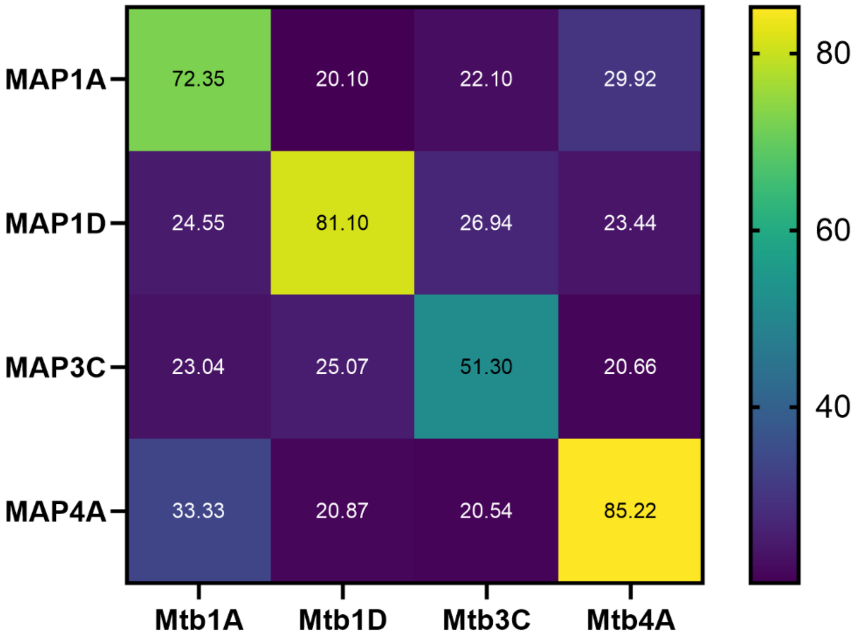

Supplementary Figure S9| Percentage identity matrix of selected Mce proteins between MAP and *M. tuberculosis.* Protein sequences for selected Mce proteins were aligned against each other to compare the percentage similarity between MAP and *M. tuberculosis* derived Mce using Clustal Omega 2.1. Percentage similarities are plotted as a heat map. The following sequences were used: Mtb1A (Rv0178); Mtb1D (Rv0172); Mtb3C (Rv1968); Mtb4A (Rv3499c); MAP1A (MAP0164); MAP1D (0161); MAP3C (MAP1654); MAP4A (MAP3204).

Antibody/staining agent	Concentration	Manufacturer Number
Anti-His*	1μg/mL	RB-10-0002-100
Anti-Dnak*	1μg/mL	A207645
Anti-E. coli	10 μg/mL	ab137967
Anti-Rabbit IgG H+L*	1μg/mL	06/2019
Anti-Mouse IgG H+L*	1 μg/mL	06/2016
Phalloidin 488	66 μM	A12379
Anti-Rabbit 594	10 μg/mL	Z25307

Supplementary Table S1| A table outlining the antibodies used in this paper and the corresponding concentration. *Indicates antibodies used for Western Blots.
